# Dietary gluten and the development of type 1 diabetes

**DOI:** 10.1007/s00125-014-3265-1

**Published:** 2014-05-29

**Authors:** Julie C. Antvorskov, Knud Josefsen, Kåre Engkilde, David P. Funda, Karsten Buschard

**Affiliations:** 1 The Bartholin Institute, Rigshospitalet, Ole Maaløes Vej 5, section 3733, Copenhagen, Denmark; 2 Department of Immunology and Gnotobiology, Institute of Microbiology, Academy of Sciences of the Czech Republic, Prague, Czech Republic

**Keywords:** Coeliac disease, Diet, Gluten, Gluten-free, Type 1 diabetes

## Abstract

Gluten proteins differ from other cereal proteins as they are partly resistant to enzymatic processing in the intestine, resulting in a continuous exposure of the proteins to the intestinal immune system. In addition to being a disease-initiating factor in coeliac disease (CD), gluten intake might affect type 1 diabetes development. Studies in animal models of type 1 diabetes have documented that the pathogenesis is influenced by diet. Thus, a gluten-free diet largely prevents diabetes in NOD mice while a cereal-based diet promotes diabetes development. In infants, amount, timing and mode of introduction have been shown to affect the diabetogenic potential of gluten, and some studies now suggest that a gluten-free diet may preserve beta cell function. Other studies have not found this effect. There is evidence that the intestinal immune system plays a primary role in the pathogenesis of type 1 diabetes, as diabetogenic T cells are initially primed in the gut, islet-infiltrating T cells express gut-associated homing receptors, and mesenteric lymphocytes transfer diabetes from NOD mice to NOD/severe combined immunodeficiency (SCID) mice. Thus, gluten may affect diabetes development by influencing proportional changes in immune cell populations or by modifying the cytokine/chemokine pattern towards an inflammatory profile. This supports an important role for gluten intake in the pathogenesis of type 1 diabetes and further studies should be initiated to clarify whether a gluten-free diet could prevent disease in susceptible individuals or be used with newly diagnosed patients to stop disease progression.

## Type 1 diabetes and coeliac disease

**Fig. 1:**
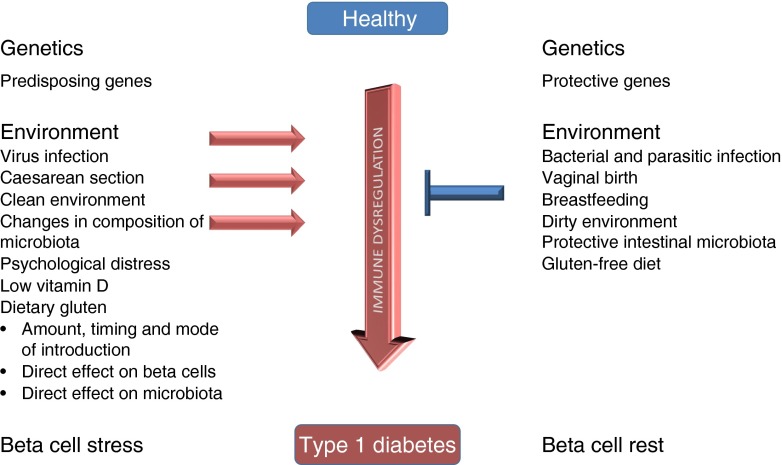
Dietary gluten affects the development of type 1 diabetes. The influence of genetic predisposition, different environmental factors and dietary gluten on disease pathogenesis. Type 1 diabetes is a multifactorial disease, the development of which is dependent on genetic as well as environment factors, which alone or together affect immune balance, resulting either in protection against or susceptibility to disease development

Type 1 diabetes incidence has increased over the last two decades, especially in children under the age of 5 [1]. Type 1 diabetes is a multifactorial disease in which the genetic background is essential, but not sufficient for causing the disease. Type 1 diabetes incidence has been rising more rapidly than can be accounted for by genetic changes, thus emphasising the influence of environmental factors [2]. Different environmental factors could play a role in type 1 diabetes susceptibility: cereal proteins, cow’s milk proteins, low vitamin D, enteroviruses, changes in the composition of gut microbiota and stressful life events. Further, the ‘hygiene hypothesis’ suggests that exposure to a large number of infections early in life prevents the development of autoimmunity through appropriate priming of the adaptive immune system [3] (Fig. 1).

Coeliac disease (CD) is an inflammatory intestinal disease with autoimmune features triggered by exposure to dietary gluten and related cereal proteins. The proteins induce an inflammatory immune response in the intestine; their withdrawal results in disease remission. The intestinal inflammation can result in complete destruction of the intestinal epithelium with crypt hyperplasia, loss of the villous structure and infiltration of lymphocytes, with consequent malabsorption of nutrients, vitamins and minerals [4]. Screening studies indicate that CD has a high prevalence (≈1%) in many Western countries and that a large group of people have undetected CD, which has led to the concept of a ‘coeliac iceberg’, depicting different undiagnosed, silent forms of CD [5].

The pathogenesis is initiated when gluten peptides cross the intestinal epithelium. The peptides can be deamidated or crosslinked by the enzyme tissue transglutaminase (tTG). Deamidation introduces a negative charge into the gluten peptides, increasing their binding affinity to HLA-DQ2 or HLA-DQ8 on antigen-presenting cells (APCs), and thereby increasing the possibility of reaching the threshold required to prime gluten-reactive T cells. The intestinal CD4^+^ T cell response is directed against many different epitopes in the gluten proteins. However, the T cell epitopes tend to cluster in proline-rich regions of the gluten proteins [4]. Of particular interest is the observation that nearly all T cell lines from adult CD patients recognise the same 33-mer gliadin peptide. It contains six HLA-DQ2-binding and T cell-stimulatory epitopes and is resistant to intestinal digestion [6]. CD is moreover characterised by IgA and IgG autoantibodies directed against tTG, by intestinal activation of T helper (Th)17 cells, CD8^+^ T cells, γδ T cells, natural killer (NK) cells and dendritic cells (DCs) [7–9], and by the direct effect of gluten on intestinal enterocytes [10].

## The association between CD and type 1 diabetes is well established

Type 1 diabetes and CD share a similar genetic background, with high susceptibility associated with the HLA-DQ2/DQ8 genotype. Several other genes are also implicated in susceptibility to both diseases, while up to 15 risk alleles contribute to both diseases [11]. This is at least part of the explanation as to why there is a high prevalence of patients with both diseases. Thus, studies have revealed an average CD prevalence of 2–12% among children with type 1 diabetes [12, 13], and among patients diagnosed with both type 1 diabetes and CD, the majority of the patients were diagnosed with type 1 diabetes before CD, probably due to the diabetes-protective effect of a gluten-free diet [14].

## Dietary gluten

The taxonomic relationship of cereals classifies them into different subfamilies, where wheat, rye and barley are in the same tribe. Wheat has highly viscoelastic properties, which allow dough to form when wheat flour is mixed with water. This viscoelastic network is called gluten. The major components of gluten are storage proteins called prolamins, which can be separated into two groups: gliadins (α-, γ- and ω-gliadin) and glutenins. The prolamin storage proteins in wheat, rye and barley differ from other cereals' storage proteins in the following ways (Table 1): they are present in higher amounts, have a higher molecular mass and contain higher proportions of proline and glutamine. Finally, the prolamins in wheat, rye and barley are insoluble in water, because of the presence of highly hydrophobic repeated sequences [15, 16].

**Table 1:** Gluten proteins differ from other cereal proteins

Property	Wheat, rye, barley	Other cereals (e.g. oats, rice, maize)
Level of prolamins	40–50%	10% (in oats)^a^
Molecular mass	30–90 kDa	10–16 kDa (in rice)^a^
Amino acid sequence	↑ Proline	↑ Leucine
	↑ Glutamine	↑ Alanine (in maize)^a^
Repeats	PQQPFPQQ	VLPA or FNQLA
	(90%)	(25% in maize)^a^

^a^Representative for non-gluten-containing cereals

Thus, the storage proteins in wheat, rye and barley have several properties that distinguish them from other cereal proteins. So far, most studies have focused on the gliadins and glutenins of wheat gluten, but because of the similar structures of rye and barley, many of the results can also be applied to these cereals.

## Gluten peptides show resistance to enzymatic processing in the intestine

The normal digestive breakdown of dietary proteins ensures that peptides are efficiently hydrolysed into amino acids and di- or tripeptides before they are transported across the intestinal epithelium. This process ensures that the proteins are rendered harmless, because di- and tripeptides cannot initiate an adaptive immune response. As mentioned, the amino acid sequences of gluten and related proteins from rye and barley are very rich in proline residues, which render them resistant to enzymatic processing at two levels: at the gastric/pancreatic level and at the brush-border peptidase level [17]. The slow cleavage rate implies that there could be an intestinal, luminal accumulation of gliadin peptides following a gluten-containing meal. In vitro experiments showed that prolonged treatment of intact α2-gliadin with gastric and pancreatic enzymes and brush-border membrane fractions resulted in a stable proteolytic fragment, the 33-mer peptide (LQLQPFPQPQLPYPQPQ-LPYPQPQLPYPQPQPF). While control dietary peptides were rapidly proteolysed, the 33-mer peptide remained intact. Homologue sequences to the 33-mer were found in rye and barley, but not in oats or other cereals [6]. Thus, proteins from wheat, rye and barley can be expected to be comparably resistant to proteolysis. Ingestion of gluten is therefore likely to result in sustained, high intestinal concentrations of non-degradable gluten peptides.

## Gluten and the intestinal microbiota

It has been reported that microbial enzymes are capable of degrading gluten proteins [18]. Data suggest that CD patients have higher levels of gliadin-metabolising enzymes with bacterial origin than do healthy controls. It has been suggested that gliadin proteolysis by intestinal bacteria could initiate CD, due to the formation of immunogenic gluten peptides [19]. The possible involvement of the intestinal microflora in the pathogenesis of CD is supported by studies showing that CD patients exhibit changes in the composition of the duodenal microbiota of the gut [20]. On the other hand, intragastric application of gluten to AVN rats (an animal model of CD) at birth induced features of gluten enteropathy [21]. Such results were also reproducible in germ-free rats, suggesting direct activation of intestinal immune responses by gluten proteins [22].

Observations that specific-pathogen-free (SPF) animal facilities (e.g. positively defined SPF microflora, such as altered Schaedler microflora), antibiotic decontamination and re-derivation of the breeding nucleus facilitate high diabetes incidence in NOD mice, show the role of intestinal microflora in type 1 diabetes development [23]. An early study in BioBreeding (BB) rats documented that treatment with fusidic acid [24] and vancomycin [25], both antibiotics against Gram-positive bacteria, reduce diabetes incidence. Heat-killed *Lactobacillus casei* fed to NOD mice also prevented development of diabetes [26]. Thus, clean conditions seem to increase type 1 diabetes incidence, whereas infections (including parasites) and administration of bacterial components decrease the development of the disease. Wen et al have further documented higher diabetes incidence in germ-free mice compared with SPF-reared NOD mice and have described possible innate immune mechanisms involved in the disease processes [27]. These data indicate that commensal microflora have a modifying, disease-preventive effect on type 1 diabetes.

Gluten intake directly affects the composition of the intestinal microflora, as NOD mice fed a gluten-free diet have reduced numbers of caecal bacteria and Gram-positive bacteria compared with mice fed a standard diet containing wheat proteins [28]. In a recent study by Marietta et al, a gluten-containing diet specifically increased *Bifidobacterium*, *Tannerella* and *Barnesiella* species, whereas *Akkermansia* was increased in the intestinal microflora of NOD mice fed a gluten-free diet [29].

Both intestinal microflora and diet influence penetration of type 1 diabetes in the animal models. When certain diabetes-preventive diets were first tested in gnotobiotic (microflora-defined) or germ-free conditions, it was revealed that microbiota-independent mechanisms are responsible for the protective effects of these diets; however, other diets (e.g. hydrolysed casein-based formula) have been found to have a microbiota-dependent diabetes-protective effect (D. P. Funda, unpublished results). Patrick et al showed that a diet based on cereals was a stronger promoter of type 1 diabetes than gut microbes; thus, a more significant protection from disease development was observed by feeding a hydrolysed casein-based diet than could be obtained by altering the microbiota [30]. More studies in germ-free and gnotobiotic animals are needed to clarify the mechanisms and the interplay between different dietary (not specifically gluten) and microbial factors in type 1 diabetes prevention, and to determine which diet-induced changes to the composition of commensal microflora have causative effects on the pathogenesis of type 1 diabetes.

## Dietary gluten influences the development of type 1 diabetes

Evidence of the interplay between ingested gluten and the subsequent development of type 1 diabetes has been revealed by studies in humans and animals.

In 1999 we demonstrated that a gluten-free, non-purified diet largely prevented diabetes onset in NOD mice, as following a gluten-free diet for 320 days reduced the incidence of diabetes from 64% in control mice to 15% in mice on the gluten-free diet [31] with a further incidence reduction to 6% in mice never exposed to gluten either in the uterus or in diet [32]. The highest incidence of type 1 diabetes in experimental animal models is found in animals on a wheat-based diet [33], and a cereal-based diet promotes type 1 diabetes development in both NOD mice and BB rats [34, 35]. This suggests a clear correlation between gluten intake and increased diabetes susceptibility.

In a preliminary study in humans, the autoantibody titres did not show significant changes in response to a gluten-free diet. However, the insulin response in a glucose tolerance test increased in 12 out of 14 patients on the gluten-free diet. After returning to a normal diet, the acute insulin response decreased in ten out of 13 patients. Furthermore, insulin sensitivity, measured as HOMA-IR, improved on the gluten-free diet and subsequently decreased after 6 months back on the normal diet [36]. The effect of a gluten-free diet for 1 year was also investigated in seven children predisposed to type 1 diabetes who tested positive for beta cell autoantibodies. The gluten-free diet did not influence the autoantibody titres [37]. This is in contrast to a recently reported study of a 5-year-old boy diagnosed with classical type 1 diabetes without CD. The patient was introduced to a gluten-free diet, resulting in a reduction in HbA_1c_ from 7.8% to 5.8–6.0% without insulin therapy. Fasting blood glucose was maintained at 4.0–5.0 mmol/l. At 16 months after diagnosis the fasting blood glucose was 4.1 mmol/l, and after 20 months he was still without daily insulin therapy, suggesting that the gluten-free diet prolonged remission in this patient with type 1 diabetes [38]. In studies of patients with both CD and type 1 diabetes, a gluten-free diet mediated clinical improvements, such as increases in weight, height, haemoglobin level, corpuscular volume and diabetic control [13, 39]. However, other studies found no effect [40, 41]. The findings indicate that a gluten-free diet may have a beneficial effect on the preservation of beta cell function in high-risk individuals. The mechanism by which gluten's removal from the diet could improve insulin secretion is, however, unclear.

The studies mentioned above investigated individuals positive for beta cell autoantibodies, which implies an already initiated immune response against the islets. However, a large human intervention trial is needed to investigate possible preventive effects of a permanent gluten-free diet in genetically predisposed individuals.

## A subset of type 1 diabetic patients has an abnormal immune response to gluten

Gluten challenge has been used in CD patients to assess their mucosal immune response to gluten, but has also been used as a tool to study an abnormal immune reactivity to gluten in type 1 diabetic patients. In a study of 19 type 1 diabetic children, 20% reacted to rectal gluten challenge by lymphocyte infiltration in the epithelium and the underlying lamina propria. The patients all had HLA-haplotypes associated with CD, but normal mucosal histology and tTG antibody-negative serology [42]. Recently, it was shown that 20 out of 42 type 1 diabetic patients (tTG antibody-negative) had increased proliferative T cell response (peripheral blood mononuclear cells) to wheat proteins, with production of proinflammatory cytokines, such as IFNγ, IL-17A and TNF. Interestingly, this response to gluten was not explained by CD-associated haplotypes, which indicates that the proinflammatory response to wheat proteins is not only present in CD patients [43]. This study is supported by earlier findings of an increased T cell response to gluten in 24% of newly diagnosed type 1 diabetic patients [44]. The results show that an abnormal mucosal immune response to gluten is present in at least a subset of type 1 diabetic patients. The association may simply reflect the common genetic background of CD and type 1 diabetes; however, the studies reporting a specific immune response to gluten in type 1 diabetic patients without CD-associated HLA-haplotype suggest a direct, diabetes-specific effect of gluten.

Besides the effect of gluten proteins on the development of type 1 diabetes, other components of wheat, such as wheat storage globulin (Glo-3A), may also be associated with disease [45]. However, Glo-3A antibody levels did not differ in a recent study comparing islet-autoantibody-positive children with controls [46].

These studies support the idea that gluten, and perhaps other wheat proteins, influence the development of type 1 diabetes.

## The diabetogenic effect of gluten can be modified

Several different factors affect the diabetogenic potential of gluten: the *amount* of ingested gluten, and the *timing* and *mode* of gluten introduction.

The dose or *amount* of ingested gluten was earlier found to be important in maintaining a tolerant immune response to gluten. NOD mice fed a diet four times higher in gluten content than the standard diet showed protection from type 1 diabetes at the same level as the gluten-free diet [32]. This finding is supported by studies showing dose-dependent effects of gluten on type 1 diabetes development in NOD mice, where high wheat concentrations returned diabetes incidence to the same level as in control mice [47].

Two prospective cohort studies (BABYDIAB and Diabetes Autoimmunity Study in the Young [DAISY]) have established a connection between the *timing* of early exposure to gluten and the risk of developing islet-specific antibodies. The BABYDIAB study found that children exposed to gluten-containing cereals before the age of 3 months have an increased risk (HR 4.0) of developing islet autoantibodies, compared with children who only received breast milk during the same period. Children receiving gluten after the age of 6 months did not have an increased risk of developing islet autoantibodies [48]. The DAISY study found that children exposed to both non-gluten-containing and gluten-containing cereals before the age of 3 months (HR 4.32), or after 7 months (HR 5.6), had an increased risk of islet autoimmunity compared with children first exposed to gluten between the ages of 4 and 6 months [49]. Moreover, the DAISY study showed that if cereals were introduced while the child was still being breastfed, the risk of developing islet antibodies was reduced [49]. Both studies found that early introduction of foods containing gluten or other cereals before the age of 3 months is associated with an increased risk of islet autoimmunity in childhood. This reflects that there may be a ‘time window’ for gluten introduction, that best allows tolerance to be achieved [50], and that the timing of the first exposure to gluten may influence overall immune tolerance to food antigens [51]. However, a delayed timing of first gluten exposure (12 vs 6 months) in infants was tried in a dietary intervention trial including 150 infants with a first-degree family history of type 1 diabetes and a risk HLA genotype. During the first 3 years of age there was no significant difference between the early and late exposure groups according to the risk for developing type 1 diabetes [52]. These results are supported by an earlier finding showing that time for gluten introduction had no influence on development of diabetes-related autoantibodies in non-diabetic children [53]. However, the latter study does not define the exact time point for gluten introduction nor does it record simultaneous breastfeeding. The importance of timing of first exposure to solid food is not only restricted to gluten exposure, as it has also been found that early introduction of root vegetables, by the age of 4 months, is associated with increased risk for beta cell autoimmunity [54], indicating that oral tolerance induction towards different food antigens could play an important role for the risk of type 1 diabetes development.

The *mode* of gluten introduction has also been shown to influence gluten's diabetogenic effect. The development of diabetes in BB rats is modified according to the diet they are fed during and after weaning. When gluten is introduced to BB rats after weaning, it seems to be a potent inducer of diabetes. On the other hand, if gluten is introduced early in BB rat neonates, while they are still being exposed to lactation, the diabetes incidence is significantly reduced [55]. This supports the previously mentioned result found in the DAISY study and is consistent with recent findings on the modification of gluten intake in NOD mice, where early avoidance of gluten immediately after weaning delays diabetes onset and is associated with reduced insulin autoantibodies and insulitis [56]. This implies that modifying exposure to gluten can influence its diabetogenic potential.

Moreover, the importance of amount, timing and mode of gluten introduction is supported by a study investigating differences in infant diet after an increase in the incidence of CD was observed in Sweden between 1985 and 1987. The annual incidence rate of CD in children below 2 years of age increased fourfold in this period, followed by a sharp decline to the previous level. The rise seemed to be the result of changes in the infant diet: an increase in the amount of gluten consumed, the postponement of gluten introduction from 4 to 6 months of age, and the interruption of breastfeeding when gluten was introduced [57]. The possible protection from CD [58] and type 1 diabetes associated with breastfeeding [59] could be explained by the immune-modulating effect of breastfeeding on the developing cellular immune system.

This implies that differences in gluten introduction can have an impact on gluten's diabetogenic potential, and thus, on its effect on the development of CD and type 1 diabetes.

## The intestinal immune system is involved in the pathogenesis of type 1 diabetes

Evidence points toward the involvement of the intestinal immune system in the development of type 1 diabetes and studies suggest that pancreas-infiltrating autoreactive T cells may be activated in the gut-associated lymphoid tissue (GALT) (Text box: The intestinal immune system is involved in the pathogenesis of type 1 diabetes).

**Table:** 

**The intestinal immune system is involved in the pathogenesis of type 1 diabetes**
**Animal studies** Diabetogenic T cells are initially primed in the gut [65] Islet-infiltrating T cells express gut-associated homing receptor [62] Mesential lymphocytes transfer diabetes to healthy mice [64] Prediabetic BB rats show intestinal morphological and immunological changes [68, 69, 95, 96] Diabetes-prone animals have increased intestinal permeability [74, 76] **Human studies** T cells derived from diabetic pancreas tissue adhere to mucosal endothelium [66] GAD-reactive lymphocytes express gut-associated homing receptor [67] Immunological activity in the small intestine is seen in diabetic patients without CD [60, 61] Gastrointestinal alterations are found in type 1 diabetic patients [70–72] Type 1 diabetic patients have high zonulin levels, associated with increased gut permeability [75]

Paediatric patients with type 1 diabetes, but without signs of CD, had increased expression of MHC class II antigens and intercellular adhesion molecule 1 (ICAM-1) on the intestinal epithelium [60, 61]. The jejunal specimens contained higher concentrations of IFNγ- and TNFα-positive cells, and had a higher density of IL-1α- and IL-4-positive cells in the lamina propria, than those of healthy controls. While IL-1α is a proinflammatory cytokine secreted by monocytes and epithelial cells during intestinal inflammation, IL-4 is a Th2 cytokine, known to enhance epithelial permeability [61]. Interestingly, these findings were not restricted to patients carrying HLA-DQ2, suggesting an intestinal inflammatory response in type 1 diabetic patients, independent of a CD-associated genotype. Therefore, it seems that the gut immune system is activated in type 1 diabetic patients, and that activation is not only due to the shared genotype with CD patients.

In NOD mice, lymphocytes infiltrating the islets, especially during the prediabetic phase, express the GALT-specific α4β7 integrin [62], and antibodies blocking the α4β7 integrin prevent diabetes [62, 63]. This suggests a role for the intestinal immune system in the early phases of diabetes. Moreover, it has been shown that mesenteric lymphocytes transfer diabetes from NOD mice to non-diabetic mice, which indicates that diabetogenic T cells are activated in the mesenteric immune system before infiltrating the pancreas [64]. Similarly, lymphocytes with diabetogenic potential isolated from 3-week-old NOD mice were found in gut-associated lymph nodes [65]. This suggests that the initial priming of diabetogenic cells takes place in the gut and that further amplification of the autoimmune response may occur in the pancreas-associated lymph nodes (PLN) [65]. Human studies also support the hypothesis that autoreactive T cells may originate from the intestinal immune system, as lymphocytes from diabetic pancreases were found to adhere specifically to mucosal and pancreatic endothelium [66] and GAD-reactive T cells from patients with type 1 diabetes were shown to express α4β7 integrin [67].

These findings suggest that the gut immune system is activated in patients with type 1 diabetes, and that the pancreas and the gut share the same lymphocyte recirculation pool. This supports the notion that autoreactive T cells infiltrating the pancreas could be activated in GALT. It is not clear, however, to what extent these changes are attributable to gluten.

## Gastrointestinal alterations in type 1 diabetes

Changes in intestinal morphology and permeability, which may or may not involve gluten, have been recognised both in animal models of type 1 diabetes and in human patients.

BB rats have several features suggesting intestinal malfunction. These include increased intestinal permeability, changes in mucosal crypt depth, massive epithelial cell proliferation, lymphocyte infiltration, and proinflammatory activity, before the development of both insulitis and diabetes [68, 69]. Human studies also support the notion that gastrointestinal changes precede the onset of type 1 diabetes. In a study of patients with islet autoimmunity at different stages all patients showed an increase in intestinal permeability in a lactulose–mannitol test [70]; alterations in intestinal barrier structure and function in non-coeliac type 1 diabetic patients have also been described [71]. Likewise, HLA-DQ2-positive paediatric diabetic patients had higher intestinal permeability, which could facilitate contact of food antigens with the mucosal immune system [72]. This implies that these patients may be more prone to developing abnormal immune responses to common food antigens. It is also consistent with the development of CD, where changes in the intestinal barrier function and tight junction structure of the jejunum of children with CD take place early on in the disease development [73]. The findings point to the crucial role intestinal barrier functions play in relation to both CD and type 1 diabetes development and, furthermore, suggest that a primary intestinal defect could exist in patients with type 1 diabetes.

The importance of changes in intestinal permeability is further supported by findings regarding changes in zonulin, a protein that opens intestinal tight junctions. In the diabetes-prone BB rat, intestinal, intraluminal zonulin levels are elevated 35-fold compared with levels in the diabetes-resistant BB rat [74]. Likewise, 70% of type 1 diabetes at-risk subjects with elevated autoantibodies but no established disease had increased serum zonulin levels [75]. Blocking the zonulin receptor reduces diabetes incidence by 70% [74] and restoration of the impaired intestinal barrier contributes to the prevention of type 1 diabetes in BB rats [76]. Thus, upregulation of zonulin, with consequent increased intestinal permeability, precedes the onset of type 1 diabetes. The latter is a direct effect of gliadin, which binds to the chemokine receptor chemokine (C-X-C motif) receptor 3 (CXCR3), leading to myeloid differentiation factor 88 (MyD88)-dependent zonulin release in enterocytes [77], but factors other than gluten could influence intestinal permeability in both animal models of type 1 diabetes and patients.

These studies show that changes in intestinal morphology and permeability possibly precede development of type 1 diabetes.

## The effect of dietary gluten on the immune system has not been clarified

The mechanisms by which dietary gluten influences the immune system have not been well characterised, although some studies have been performed (Text box: In vivo and in vitro studies provide evidence for a direct effect of gluten on the immune system).

**Table:** 

**In vivo and in vitro studies provide evidence for a direct effect of gluten on the immune system**
**Effect of gluten intake (in vitro studies)** Macrophages: proinflammatory cytokine production; NO production [85, 86, 97] Dendritic cells: upregulation of MHCII; maturation markers; co-stimulatory molecules; TLRs; cytokine and chemokine production [7, 8, 84] **Effect of gluten intake (in vivo studies)** NOD mice show Th1/Th17 cytokine bias in the intestine [78, 98] NOD mice show increased activated intestinal CD4^+^ T cells, DCs and Th17 cells [98] BB rats show a Th1 cytokine bias in MLN [79] and intestine [55] BB rats show a Th1 cytokine pattern in islet infiltrate [83] Proportional changes in regulatory T cell subsets in BALB/c mice [81] Increased number of Th17 cells in PLN in BALB/c mice [81] Inflammatory cytokine pattern in FOXP3^−^ and FOXP3^+^ T cells in BALB/c mice [82] Innate immune activation [99]

First, in NOD mice, gluten intake promotes a Th1 cytokine bias in the small intestine, compared with a semi-purified hypoallergenic diet [78]. This was confirmed in a study in young BB rats showing that a wheat-based diet induces a proinflammatory Th1 bias in the mesenteric lymph nodes (MLN), with a high proportion of IFNγ^+^ CD4^+^ T cells that proliferate specifically in response to wheat protein antigens. In addition, abundance of the Th2 cytokine-specific transcription factor GATA3 was reduced, while no change was seen in the expression of the Th1 cytokine-specific transcription factor T-bet. This suggests that the MLN display a Th1 bias as a result of the Th2 deficit. The Th1 cytokine bias existed as early as 1 week before weaning, implying that the MLN cells were primed towards a Th1 phenotype, prior to pancreatic inflammation [79]. Conversely, BB rats fed a cereal-based diet exhibited a decreased IFNγ/TGFβ ratio in the gut [55]. This could imply that gluten's ability to induce a Th1 cytokine profile in the gut of CD patients [80] also occurs in animal models of type 1 diabetes. Recently, we published data showing that gluten intake in BALB/c mice led to a decreased proportion of γδ T cells in all lymphoid compartments studied, while the number of Th17 cells, which are associated with the development of autoimmunity, increased substantially in PLN [81]. Furthermore, the gluten-containing diet changed both forkhead box P3 (FOXP3)^−^ T cells and FOXP3^+^ T cells, to a more inflammatory cytokine profile, with higher levels of IL-17, IL-2, IL-4 and IFNγ. In contrast, the gluten-free diet induced an anti-inflammatory cytokine profile, with higher proportions of TGFβ^+^FOXP3^−^ T cells in all of the tested lymphoid tissues and higher IL-10 expression within non-T cells in the spleen [82]. Besides the intestinal Th1 cytokine bias, a cereal-based diet also promoted a Th1 cytokine pattern (high IFNγ and low IL-10 and TGFβ) in islet infiltrates. Conversely, BB rats fed a hydrolysed, casein-based, semi-purified diet had fewer islet-infiltrating cells, low IFNγ and high levels of TGFβ. The non-cereal-based diet thus protected BB rats from developing diabetes by inducing a non-inflammatory cytokine pattern in the pancreas [83]. It is not completely understood whether the immunological changes are exclusively due to the effect of gluten or secondary to gluten-induced changes in, for example, intestinal microbiota.

Interestingly, gluten has also been shown to directly stimulate APCs. DCs derived from the bone marrow of BALB/c mice were exposed to chymotrypsin-treated gluten, which induced DC maturation with upregulation of MHC II, co-stimulatory molecules (CD86, CD40, CD54) and high production of MIP-2 and keratinocyte-derived cytokine (KC). DCs exposed to 100 μg/ml gluten showed comparable effects to DCs exposed to 10 ng/ml lipopolysaccharide, which emphasises the stimulating capacity of gluten [7]. Moreover, α-chymotrypsin-digested gliadin stimulated the expression of Toll-like receptors (TLRs) 4, 7 and 8, and the secretion of IFNα from bone marrow-derived DCs in transgenic HLA-DQ8 mice [84]. Human monocyte-derived DCs are also matured by gliadin. Stimulation leads to enhanced expression of CD80, CD83 and CD86, plus upregulation of HLA-DR, accompanied by an increased secretion of IL-6, IL-8, IL-10, TNFα, monocyte chemotactic protein (MCP-1) and MCP-2. After gliadin stimulation, the DCs showed reduced endocytosis and an improved capacity to stimulate the proliferation of allogeneic T cells [8]. Besides having an effect on DCs, gliadin peptides also induce murine peritoneal macrophages to express proinflammatory cytokine genes such as TNFα, IL-12 and IL-15 [85] and NO production [86]. The cytokine production was dependent on MyD88, which is a key adapter molecule in the TLR/IL-1R signalling pathway [85]. The gluten epitopes recognised by DCs and macrophages, and the exact mechanisms of recognition, remain to be clarified. However, the direct effect of gluten on APCs emphasises gluten's immunogenic properties, and shows that gluten peptides have the capacity to stimulate APCs to deliver co-stimulatory signals to T cells.

Interestingly, it has been shown that specific gluten peptides (e.g. p31-43) have a direct effect on the innate immune system [87]. These gluten peptides induce expression of the innate cytokines IL-15 and IFNα in the intestinal lamina propria [10, 88], altering the tolerogenic phenotype of the DCs. Moreover, these peptides also have the capacity to directly activate epithelial cells in the gut by upregulation of non-classical MHC class-I molecules (HLA-E), MIC-A and MIC-B, and induce IL-15 secretion [89, 90]. MIC serves as a ligand for the NK receptor NKG2D, which is expressed on the intraepithelial CD8^+^ T lymphocytes. The cells become so-called lymphokine-activated killer cells with NK-like cytolytic function, that is, they have the ability to kill target cells independent of T cell receptor ligation. These cells induce direct cytolysis of enterocytes via NKG2D–MIC engagement in patients with CD [91]. Dietary gluten is thus found to induce a Th1 cytokine bias in the gut and islet infiltrates in NOD mice and BB rats, and it seems to induce innate cell activation and maturation of APCs. Recently, it has been suggested that some of the effects of gluten proteins are not mediated by the proteins themselves, but by wheat amylase trypsin inhibitors, which furthermore activate TLR4 [92].

## Direct effects of gluten on pancreatic beta cells

Gliadin not only modulates the immune system but also affects the target cells: the pancreatic beta cells. This was recently shown in vitro and in vivo using gluten digested with a range of digestive enzymes, as well as a 33-mer gliadin peptide (residues 57–89 of alpha 2-gliadin). In vitro, both stimuli increased the long-term (24 h), but not the short-term (30 min), insulin secretion. It was possible to demonstrate that the effect was due to closure of the ATP-sensitive K-channels, by directly affecting the channels. In vivo, a long-term effect was also seen. NOD mice, which were injected with gliadin digest, obtained higher body weights as adults, probably reflecting the higher insulin secretion from the islets [93]. Beta cell stress can contribute to diabetes development through increased antigen expression, whereas a reduction of diabetes incidence is seen in animal models with early prophylactic insulin treatment of diabetes-prone animals [94].

## Conclusion

The present review describes the findings and development of our knowledge over the last decades on the connection between gluten and the pathogenesis of type 1 diabetes. The studies have primarily focused on describing incidence of type 1 diabetes in relation to the timing of introducing gluten into the diet, and the influence of gluten on the intestinal flora and the immune system. Most important is to evaluate the effect of a gluten-free diet on human type 1 diabetes: in this regard, a promising case has been published. In the future, the balance between the innate and adaptive immune systems must be clarified. The research field covering gluten, diet and type 1 diabetes has proven surprisingly interesting and requires further attention.

## Abbreviations

APC: Antigen-presenting cell
BB: BioBreeding
CD: Coeliac disease
DAISY: Diabetes Autoimmunity Study in the Young
DC: Dendritic cell
FOXP3: Forkhead box P3
GALT: Gut-associated lymphoid tissue
MLN: Mesenteric lymph nodes
MyD88: Myeloid differentiation factor 88
NK: Natural killer
PLN: Pancreas-associated lymph nodes
SPF: Specific-pathogen-free
Th: T helper
TLR: Toll-like receptor
tTG: Tissue transglutaminase
